# Photodeprotectable *N*-Alkoxybenzyl Aromatic Polyamides

**DOI:** 10.3390/polym9070246

**Published:** 2017-06-24

**Authors:** Kenichi Iwashita, Hironobu Katoh, Yoshihiro Ohta, Tsutomu Yokozawa

**Affiliations:** 1 Photosensitive Material R&D Department, Hitachi Chemical Co. Ltd., 4-13-1 Hitachi, Ibaraki 317-8555, Japan; 2 Department of Materials and Life Chemistry, Kanagawa University, Rokkakubashi, Kanagawa-ku, Yokohama 221-8686, Japan; kato0614cute@icloud.com (H.K.); y-ohta0112@kanagawa-u.ac.jp (Y.O.)

**Keywords:** *N*-protected polyamide, polycondensation, photo acid generator, photodeprotection

## Abstract

*N*-alkoxybenzyl aromatic polyamides were synthesized by polycondensation of *N-*alkoxybenzyl aromatic diamine with equimolar dicarboxylic acid chloride in the presence of 2.2 equiv. of pyridine at room temperature for 2 days. The obtained polyamides were mainly cyclic polymers, as determined by means of matrix-assisted laser desorption ionization time-of-flight (MALDI-TOF) mass spectrometry, and showed higher solubility in organic solvents than unprotected aromatic polyamides. Photodeprotection of *N*-alkoxybenzyl aromatic polyamide film containing photo acid generator (PAG) proceeded well under UV irradiation (5 J/cm^2^), followed by heating at 130 °C for 15 min. The nature of the polymer end groups of *N*-alkoxybenzyl aromatic polyamides was found to be crucial for photodeprotection reactivity. These polymers are promising candidates for photosensitive heat-resistant materials for fine Cu wiring formation by electroless Cu plating of high-density semiconductor packaging substrates.

## 1. Introduction

Electronic devices, such as mobile phones, tablets, and personal computers, are becoming dramatically smaller and more functionalized. Thus, there is a continuing requirement for packaging structures for semiconductors to be made smaller and thinner, and consequently further scaling-down of Cu wiring and vias in packaging substrates is needed [1]. The semi-additive process (SAP) is currently a cutting-edge technology for Cu wiring and via formation in packaging substrate manufacturing. In this process, electroless Cu plating is applied to form patterned Cu wires on the substrate. To obtain fine Cu wiring below 5 μm, sputtering Ti/Cu has been investigated [2]. CO_2_ lasers have been used for via formation to non-photosensitive heat-resistant material, while UV-YAG and excimer lasers have been studied to open vias below 50 μm in diameter [3]. However, these techniques present difficulties in removal of residues on Cu, and there is a risk of damage to Cu wiring due to ablation by the laser.

Photosensitive heat-resistant materials, such as polyimides (PI) or polybenzoxazole (PBO), exhibit excellent mechanical properties and are used in microelectronics application [4,5] to simplify via formation processing by photolithography. We have developed film-type photosensitive heat-resistant materials for high-density packaging, such as phenolic-resin-based negative tone resist containing cross-linkers and photo acid generator (PAG), and these materials provide high resolution (10 μm via for 25 μm-thick film), high adhesion to Ti/Cu sputtering seed layer, and capability to fabricate Cu wiring of less than 5 μm [6]. The sputtering method can enhance adhesion strength, but it is difficult to increase throughput and production cost is high.

Polyamides derived from diamine and dicarboxylic acid exhibit high heat-resistance, and are mainly used in high-temperature environments [7,8,9,10]. It is well known that amide linkage structure interacts with metal and metal salts such as Cu and Mn [11,12]. The adhesion strength of diallyl phthalate resin for Cu is improved by addition of polyamide [13]. Accordingly, we considered that polyamides would be promising materials to enhance adhesion strength in electroless Cu plating. However, conventional aromatic polyamides are insoluble in organic solvents because of strong intermolecular interactions based on the rigid structure and hydrogen bonding of the amide linkages. 

**Scheme 1:**
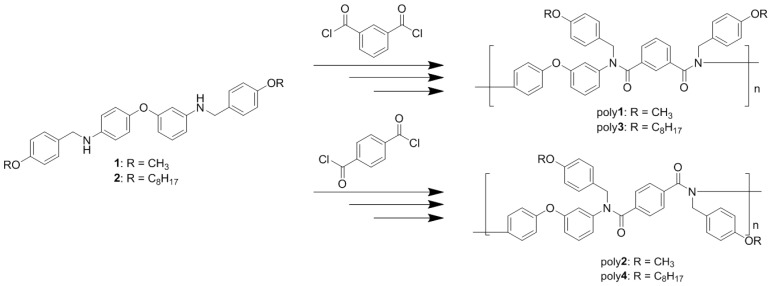
Synthesis of *N*-alkoxybenzyl aromatic polyamides, poly**1**–poly**4**, from **1** or **2** with dicarboxylic dichloride.

**Scheme 2:**
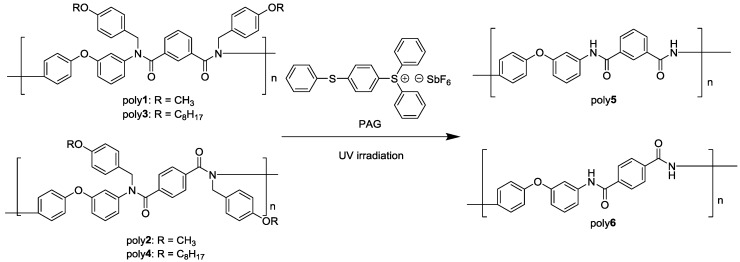
Photo-deprotection of *N*-alkoxybenzyl aromatic polyamides, poly**1**–poly**4**, with PAG.

We have reported synthesis of aromatic polyamides having an *N*-alkoxybenzyl group as a protecting group of the amide linkage [14,15]. These polyamides are soluble in several organic solvents and are converted to *N*-H polyamides by treatment with trifluoroacetic acid (TFA) at room temperature. If photodeprotection of *N*-alkoxybenzyl aromatic polyamide with PAG proceeds under UV irradiation, we could obtain promising photosensitive heat-resistant materials applicable for fine Cu wiring formation with electroless Cu plating for high-density packaging substrates. In other words, the photosensitive *N*-alkoxybenzyl aromatic polyamide would be converted to aromatic polyamide having a high adhesion property in the UV-irradiated area. In this article, we describe the synthesis of a series of *N*-alkoxybenzyl aromatic polyamides, poly**1**–poly**4**, derived from *N*-alkoxybenzyldiamines **1** and **2** and dicarboxylic acid dichlorides (Scheme 1). We further examined photodeprotection of the obtained polyamides with PAG (Scheme 2). 

## 2. Materials and Methods 

### 2.1. Measurement

^1^H NMR spectra were obtained on JEOL ECA-600 and ECA-500 (JEOL RESONANCE Inc., Tokyo, Japan) instruments operating in the pulsed Fourier-transfer (FT) mode, with tetramethylsilane (TMS, 0.00 ppm) as an internal standard. IR spectra were recorded on a JASCO FT/IR-410 (JASCO Corporation, Tokyo, Japan). Diamond attenuated total reflection (ATR) spectra were recorded on DIGLAB FTX3000MX (Bio-Rad Labo-ratories, Inc., Hercules, CA, USA). The *M*_n_ and *M*_w_/*M*_n_ values of polymers were measured on a Shodex GPC-101 (eluent: THF; calibration, polystyrene standards, SHOWA DENKO K.K., Tokyo, Japan) equipped with Shodex UV-41, Shodex RI-71S, and two Shodex KF-804-L columns (SHOWA DENKO K.K., Tokyo, Japan). MALDI-TOF mass spectra were recorded on a Shimadzu/Kratos AXIMA-CFR plus (Shimadzu/Kratos, Manchester, UK) in the reflectron ion mode by use of a laser (λ = 337 nm). Dithranol (1,8-dihydroxy-9[10H]-anthracenone) was used as the matrix for the MALDI-TOF mass measurements. 

### 2.2. Materials

Isophthaloyl chloride (IPC), terephthaloyl chloride (TPC), 3,4′-diaminodiphenyl ether (DDE), 4-methoxybenzaldehyde, and 4-octyloxybenzaldehyde were purchased from Tokyo Chemical Industry (TCI, Tokyo, Japan). Dehydrated tetrahydrofuran (dry THF, Kanto, Japan) and dehydrated dichloromethane (dry CH_2_Cl_2_, Kanto, Japan) were used as received, without purification. PAG, CPI-110A, was purchased from Sanyo Chemical Industries, Ltd, Tokyo, Japan. Other reagents were purchased from TCI or Wako Pure Chemical Industries (Wako, Japan) and used as received. Aromatic polyamides, poly**5** and poly**6** were prepared by polycondensation of 3,4′-DDE with IPC and TPC, respectively.

### 2.3. Synthesis of N-Alkoxybenzyl Aromatic Diamines ***1*** and ***2***

#### 2.3.1. Diimine **3**

A mixture of 3,4′-DDE (4.00 g, 20.0 mmol) and 4-methoxybenzaldehyde (12 mL, 100 mmol) was stirred at 70 °C for 10 min, and then poured into ethanol. The precipitated product was collected by filtration and washed with ethanol. The product was dried under vacuum overnight to give **3** (8.34 g, 96%): 106.5–110.5 °C; ^1^H NMR (600 MHz, CDCl_3_) δ 8.41 (s, 1 H), 8.35 (s, 1 H), 7.85 (d, *J* = 2.8 Hz, 2 H), 7.82 (d, *J* = 3.1 Hz, 1 H), 7.33 (t, *J* = 8.0 Hz, 1 H), 7.22 (d, *J =* 3.0 Hz, 1 H), 7.07 (d, *J* = 3.2 Hz, 1 H), 6.99–6.96 (m, 4 H), 6.92 (dd, *J* = 1.0 and 7.0 Hz, 1 H), 6.88 (dd*, J* = 1.0 and 7.5 Hz, 1 H), 6.84 (d, *J* = 2.1 Hz, 1 H), 3.87 (s, 3 H), 3.87 (s, 3 H).

#### 2.3.2. *N*,*N*′-bis(4-methoxybenzyl)diamine **1**

To a solution of **3** (3.01 g, 6.6 mmol) in dry THF (60 mL), methanol (30 mL) and NaBH_4_ (0.76 g, 27.0 mmol) were added with stirring at 0 °C. The reaction mixture was stirred at 0 °C for 21 h, and methylene chloride (CH_2_Cl_2_) was added to it. The organic layer was washed with saturated aqueous NaHCO_3_, dried over anhydrous MgSO_4_, and concentrated under reduced pressure. The obtained residue was recrystallized from toluene–hexane (1:3) to give **1** as a colorless solid (2.34 g, 80%): mp 67.5–70.0 °C; ^1^H NMR (600 MHz, CDCl_3_) δ 7.30 (d, *J* = 8.2 Hz, 2 H), 7.24 (d, *J* = 7.9 Hz, 2 H), 7.04 (t, *J* = 8.0 Hz, 1 H), 6.89–6.85 (m, 3 H), 6.60 (d, *J* = 6.0 Hz, 1 H), 6.28 (dd, *J* = 2.5 and 6.0 Hz, 1 H), 6.25 (dd*, J* = 2.5 and 6.5 Hz, 1 H), 6.20 (s, 1 H), 4.23 (s, 2 H), 4.18 (s, 2 H), 3.80 (s, 3 H), 3.80 (s, 3 H).

#### 2.3.3. Diimine **4**

Diimine **4** was synthesized in the same manner as **3** using 4-octyloxybenzaldehyde instead of 4-methoxybenzaldehyde (yield 92%): mp 96.5–101.0 °C; ^1^H NMR (600 MHz, CDCl_3_) δ 8.40 (s, 1 H), 8.30 (s, 1 H), 7.82 (d, *J* = 8.9 Hz, 2 H), 7.80 (d, *J* = 8.9 Hz, 2 H), 7.32 (t, *J* = 8.0 Hz, 1 H), 7.20 (d, *J* = 3.0 Hz, 2 H), 7.07 (d, *J* = 3.0 Hz, 2 H), 6.97–6.94 (m, 3 H), 6.92 (d, *J* = 1.0 Hz, 1 H), 6.87 (d, *J* = 1.5 Hz, 1 H), 6.84 (s, 1 H).

#### 2.3.4. *N*,*N*′-Bis(4-octyloxybenzyl)diamine **2**

Diamine **2** was synthesized in the same manner as **1** using ethanol instead of toluene–hexane (1:3) (yield 78%, yellow solid): mp 50.1–55.6 °C; ^1^H NMR (600 MHz, CDCl_3_) δ 7.28 (d, *J* = 8.2 Hz, 2 H), 7.23 (d, *J* = 8.2 Hz, 2 H), 7.04 (t, *J* = 8.0 Hz, 1 H), 6.89–6.82 (m, 6 H), 6.60 (d, *J* = 8.5 Hz, 2 H), 6.28 (d, *J* = 8.2 Hz, 1 H), 6.24 (d, *J* = 7.9 Hz, 1 H), 6.22 (s, 1 H), 1.78–1.77 (m, 4 H), 1.45–1.42 (m, 4 H), 1.34–1.25 (m, 16 H), 0.89–0.87 (m, 6 H).

### 2.4. Synthesis of N-Alkoxybenzyl Aromatic Polyamide

#### 2.4.1. Synthesis of Poly**1**

A solution of **1** (0.70 g, 1.6 mmol) and pyridine (0.3 mL, 3.5 mmol) in dry NMP (1.4 mL) was placed in a round-bottomed flask equipped with a three-way stopcock under an Ar atmosphere. Into the flask was added IPC (0.32 g, 1.6 mmol) in dry NMP (1.4 mL) via a syringe from the three-way stopcock with a stream of N_2_ at 0 °C. The reaction mixture was stirred at room temperature for 2 days. A small amount of methanol (0.5 mL) was added to quench the reaction, and the mixture was diluted with NMP (4 mL). The whole mixture was poured into water. The precipitate was collected by filtration and washed with water. The product was dried under vacuum overnight to give poly**1** as a colorless solid (0.90 g, 94%): ^1^H NMR (600 MHz, CDCl_3_) δ 7.43–7.35 (m, 1 H), 7.21–6.99 (m, 7 H), 6.91–6.82 (m, 1 H), 6.78 (d, *J* = 7.9 Hz, 2 H), 6.75 (d, *J* = 7.6 Hz, 2 H), 6.69–6.59 (m, 3 H), 6.45–6.32 (m, 4 H), 5.02–4.87 (m, 4 H), 3.71 (s, 3 H), 3.69 (s, 3 H); IR (KBr) 3449, 2925, 2854, 1648, 1610, 1512, 1485, 1440, 1381, 1302, 923, 814, 762, 725, 702, 546 cm^−1^.

#### 2.4.2. Synthesis of Poly**2**

Poly**2** was synthesized in the same manner as poly**1** using TPC instead of IPC (yield 91%): ^1^H NMR (600 MHz, CDCl_3_) δ 7.20–6.98 (m, 9 H), 6.87–6.61 (m, 9 H), 6.35–6.19 (m, 2 H), 5.11–4.84 (m, 4 H), 3.78–3.67 (m, 6 H); IR (KBr) 2925, 2854, 1648, 1610, 1512, 1485, 1460, 1379, 1303, 1249, 1212, 1175, 1109, 1033, 973, 922, 849, 761, 724, 700, 610, 543, 448, 436, 404 cm^−1^.

#### 2.4.3. Synthesis of Poly**3**

Poly**3** was synthesized in the same manner as poly**1** using **2** instead of **1** (yield 77%): ^1^H NMR (600 MHz, CDCl_3_) δ 7.43–7.31 (m, 1 H), 7.20–6.95 (m, 7 H), 6.93–6.84 (m, 1 H), 6.78 (d, *J* = 7.9 Hz, 2 H), 6.72 (d, *J* = 8.2 Hz, 2 H), 6.70–6.56 (m, 3 H), 6.52–6.32 (m, 4 H), 5.06–4.83 (m, 4 H), 3.96–3.81 (m, 4 H), 1.72 (quint, *J* = 6.9 Hz, 4 H), 1.41 (quint, *J* = 6.9 Hz, 4 H), 1.36–1.22 (m, 16 H), 0.87 (d, *J* = 6.9 Hz, 6 H); IR (KBr) 2926, 1648, 1610, 1510, 1381, 1247, 1173, 1109, 1028, 922, 810, 725, 701, 610, 539, 408 cm^−1^.

#### 2.4.4. Synthesis of Poly**4**

Poly**4** was synthesized in the same manner as poly**2** using **2** instead of **1** (yield 77%): ^1^H NMR (600 MHz, CDCl_3_) δ 7.19–7.01 (m, 9 H), 6.86–6.51 (m, 9 H), 6.39–6.21 (m, 2 H), 5.07–4.87 (m, 4 H), 3.96–3.81 (m, 4 H), 1.78–1.69 (m, 4 H), 1.47–1.39 (m, 4 H), 1.37–1.21 (m, 16 H), 0.92–0.83 (m, 6 H); IR (KBr) 3855, 3753, 3651, 2926, 2855, 1648, 1610, 1509, 1485, 1638, 1382, 1246, 1174, 1110, 1022, 972, 921, 851, 724, 701, 616, 437, 408 cm^−1^.

### 2.5. Synthesis of N-H Aromatic Polyamide

#### 2.5.1. Synthesis of Poly**5**

A solution of 3,4′-TPA (0.50 g, 2.5 mmol), lithium chloride (0.27 g, 6.4 mmol), and pyridine (0.5 mL, 5.8 mmol) in dry NMP (2.5 mL) was placed in a round-bottomed flask equipped with a three-way stopcock under an Ar atmosphere. Into the flask was added IPC (0.51 g, 2.5 mmol) in dry NMP (2.5 mL) via a syringe from the three-way stopcock with a stream of N_2_ at 0 °C. The reaction mixture was stirred at room temperature for 2 days. A small amount of methanol (0.5 mL) was added to quench the reaction, and the mixture was diluted with NMP (4 mL). The whole mixture was poured into water. The precipitate was again dissolved with NMP (15 mL) and poured into water. The precipitate was collected by filtration and washed with water. The product was dried under vacuum overnight to give poly**5** as a light yellow solid (0.82 g, 81%): ^1^H NMR (600 MHz, DMSO d_6_) δ10.47 (d, *J* =2.0 Hz, 2 H), 8.52 (t, *J* = 18.6 Hz, 1 H), 8.15 (d, *J* = 6.7 Hz, 2 H), 7.83 (d, *J* = 6.9 Hz, 2 H), 7.66 (t, *J* = 8.9 Hz, 1 H), 7.56 (d, *J* = 6.5 Hz, 2 H), 7.36–7.33 (m, 2 H), 7.10–7.08 (m, 2 H), 6.76(s, 1H); IR (KBr) 3290, 1663, 1505, 1414, 976, 839, 688, 509, 407, cm^−1^.

#### 2.5.2. Synthesis of Poly**6**

Poly**6** was synthesized in the same manner as poly**5** using TPC instead of IPC (yield 110% including small amount of NMP): ^1^H NMR (600 MHz, DMSO d_6_) δ10.45 (s, 2 H), 8.11–8.05 (m, 4 H), 7.84 (d, *J* = 5.8 Hz, 2 H), 7.56 (s, 2 H), 7.36 (s, 1 H), 7.11 (s, 2 H), 6.78 (s, 1 H); IR (KBr) 3855, 3753, 3651, 3294, 2361, 1656, 1601, 1543, 1507, 1422, 1304, 1264, 1212, 1173, 1149, 1115, 1016, 975, 841, 783, 687, cm^−1^.

### 2.6. Deprotection of N-Alkoxybenzyl Aromatic Polyamide

A mixture of poly**1** (0.01 g, 0.017 mmol) and TFA (1.0 mL) was stirred at room temperature for 2 days, and then neutralized with saturated aqueous NaHCO_3_. The polymer was collected by filtration on filter paper, and washed with CH_2_Cl_2_ and water. The product was dried under vacuum overnight. Deprotection of poly**3** was done in the same manner as that of poly**1**.

### 2.7. Evaluation of Photosensitivity

#### 2.7.1. Preparation of Photosensitive Film

A solution of *N*-alkoxybenzyl aromatic polyamide (poly**1** to poly**4**) and CPI-110A in methyl ethyl ketone (MEK) was prepared. The solution (5 wt %) was cast on an aluminum sheet and soft-baked on a hot plate at 80 °C for 5 min to afford 0.5 μm-thick thin film. 

#### 2.7.2. Evaluation of Photo-Deprotection 

The photosensitive film was exposed with 5 J/cm^2^ of UV irradiation (USHIO SP-500D, USHIO INC., Tokyo, Japan). The exposed film was heated on a hot plate at 130 °C for 15 min. Deprotection was evaluated by ATR spectroscopy.

## 3. Results and Discussion

### 3.1. Synthesis of N-Alkoxybenzyl Aromatic Diamine

**Scheme 3:**
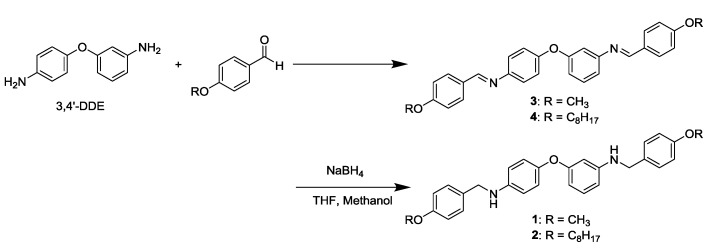
Synthesis of *N*-alkoxybenzyl aromatic diamines **1** and **2**.

We synthesized *N*-alkoxybenzyl poly(*p*-benzamide) [14] and poly(*m*-benzamide) [15] by means of chain-growth condensation polymerization of the corresponding 4-(4-alkoxybenzylamino)benzoic acid ester with a strong base, such as lithium hexamethyldisilazide. From the viewpoint of versatility, however, it would be preferable to synthesize *N*-alkoxybenzyl-protected aromatic polyamides by polycondensation of aromatic diamines having *N*-alkoxybenzyl groups with aromatic dicarboxylic acid chloride. The requisite *N-*alkoxybenzyl aromatic diamines **1** and **2** were prepared by reduction of the corresponding imine with NaBH_4_ (Scheme 3) [14]. Diamine, 3,4′-DDE, was reacted with 4-methoxy- and 4-octyloxybenzaldehyde at 70 °C for 10 min to give imines **3** and **4**, respectively, in quantitative yield. The obtained imines **3** and **4** were then reacted with 4 equiv. of NaBH_4_ at 0 °C for 21 h to afford the desired *N*-alkoxybenzyldiamines **1** and **2** in 80 and 39% yield, respectively.

### 3.2. Synthesis of N-Alkoxybenzyl Aromatic Polyamide

**Scheme 4:**
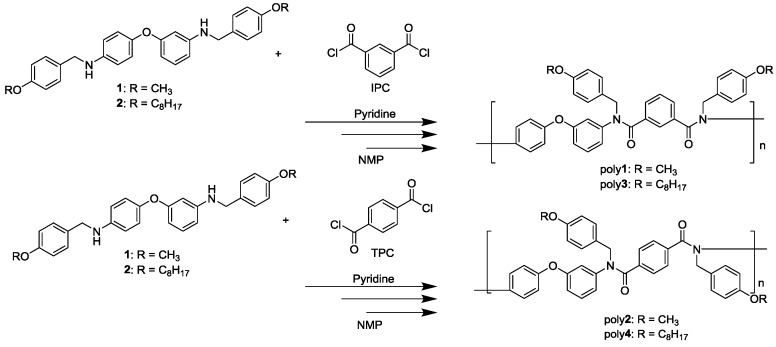
Synthesis of *N*-alkoxybenzyl aromatic polyamides.

**Figure 1:**
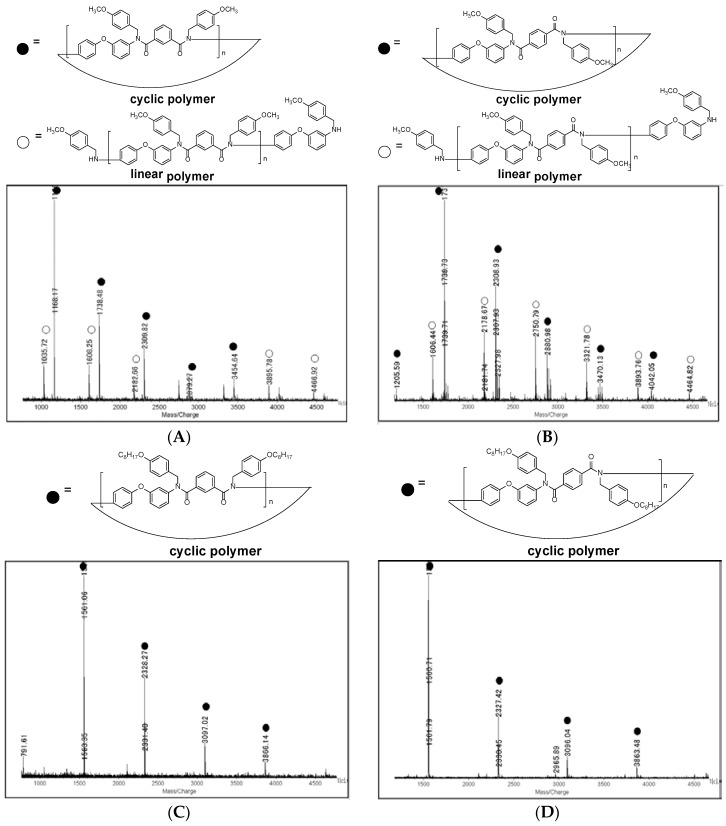
Matrix-assisted laser desorption ionization time-of-flight (MALDI-TOF) mass spectra of *N*-alkoxybenzyl aromatic polyamides: (**A**) poly**1**, (**B**) poly**2**, (**C**) poly**3**, and (**D**) poly**4**.

The polycondensation of *N*-alkoxybenzyl aromatic diamines **1** and **2** with equimolar IPC and TPC was carried out in the presence of 2.2 equiv. of pyridine in NMP at room temperature for 2 days to afford poly**1**–poly**4** (Scheme 4). Table 1 shows the molecular weight (*M*_n_ and *M*_w_) and molecular weight distribution (*M*_w_/*M*_n_) of the obtained polymers. The molecular weights of polymers obtained from diamine **1** (poly**1** and poly**2**) were lower than those of polymers from diamine **2** (poly**3** and poly**4**) (Entry 1 vs. Entry 3; Entry 2 vs. Entry 4). Impurities in **1** might have influenced the *M*_n_ of poly**1** and poly**2**. Regarding diacid chlorides, polymer from IPC possessed higher molecular weight than polymer from TPC (Entry 1 vs. Entry 2; Entry 3 vs. Entry 4) We had speculated that polycondensation of diamine with IPC would afford lower-molecular-weight polymer, compared to polycondensation with TPC, because the former polycondensation would involve easy cyclization due to the bent structure of IPC. However, polycondensation of diamine **2** and diacid chlorides only afforded cyclic polymers (poly**3** and poly**4**), irrespective of the use of IPC or TPC; the matrix-assisted laser desorption ionization time-of-flight (MALDI-TOF) mass spectra of poly**3** and poly**4** showed only one series of peaks due to cyclic polymer (Figure 1C,D). Consequently, it turned out that TPC underwent cyclization more easily, affording lower-molecular-weight polymer, than did IPC, contrary to our expectation. 

On the other hand, the MALDI-TOF mass spectra of poly**1** and poly**2** showed two series of peaks due to both the cyclic polymer and linear polymer having amino groups at both ends (Figure 1A,B). Kricheldorf et al. reported that many polycondensations afford cyclic polymers if side reactions do not take place [16,17]. Therefore, the formation of cyclic polymer and linear polymer in the polycondensation of **1** with IPC and TPC implies that a small amount of impurity in **1** presumably caused side reactions that consumed IPC and TPC, besides amide bond formation, affording linear polymer formation with amino groups at both ends.

The solubility of *N-*alkoxybenzyl aromatic polyamides poly**1**–poly**4** was studied compared to that of *N*-H polyamides poly**5** and poly**6** (Table 2). Poly**5** and poly**6** were only soluble in highly polar solvents such as DMF, NMP, and DMSO, whereas poly**1**–poly**4** were soluble in a variety of organic solvents, except for hexane and methanol. The observed differences in solubility can be accounted for by the presence or absence of intermolecular hydrogen bonding of the amide linkages in the polymer backbone. In addition, this distinctive difference in solubility between *N*-protected polyamide and *N*-H polyamide indicates that *N*-protected polyamide can serve as a photosensitive material in the presence of PAG.

### 3.3. Photodeprotection of N-Alkoxybenzyl Aromatic Polyamide

**Scheme 5:**
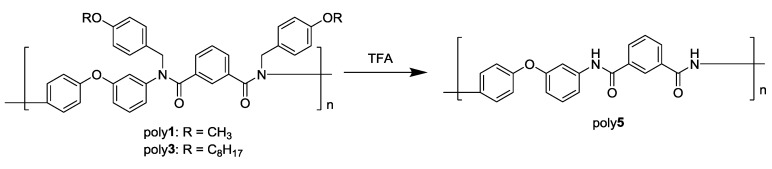
Deprotection of *N*-alkoxybenzyl aromatic polyamide.

The deprotection of *N-*alkoxybenzyl aromatic polyamides, poly**1** and poly**3**, was first conducted with TFA in homogeneous solution at room temperature for 2 days (Scheme 5). In the ^1^H NMR spectra of the product, the signals of alkoxybenzyl group had disappeared, and a broad signal assignable to a NH proton appeared at 10.5 ppm. Furthermore, the spectra of the products from poly**1** and poly**3** were identical to those of poly**5** and poly**6**, which were prepared from unprotected diamine and IPC or TPC, respectively. Accordingly, it turned out that deprotection of poly**1** and poly**3** with TFA proceeded quantitatively, and that there was no difference between the methoxybenzyl group and octyloxybenzyl group in regard to removal with TFA in homogeneous solution.

**Scheme 6:**
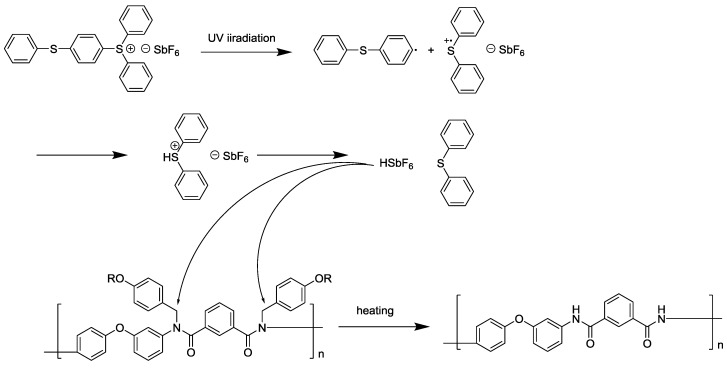
Photodeprotection of *N*-alkoxybenzyl aromatic polyamide using a photo acid generator (PAG).

**Figure 2:**
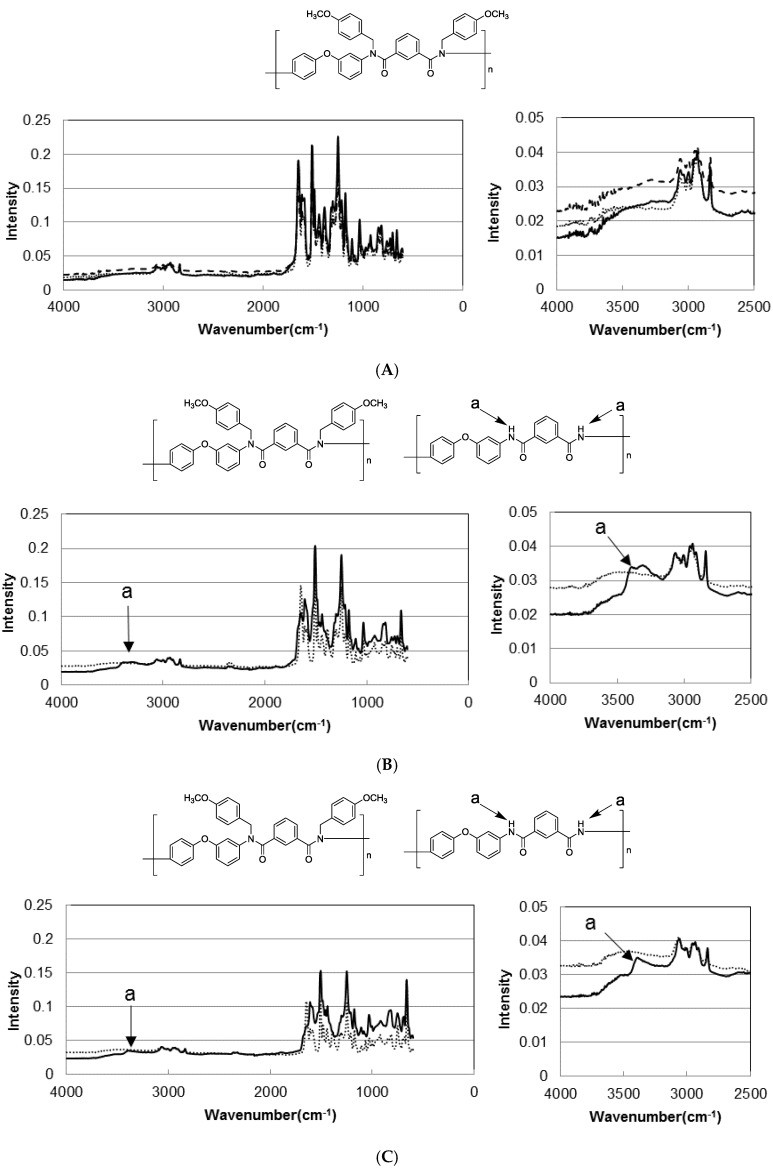
Attenuated total reflection (ATR) spectrum of photosensitive film of poly**1** and PAG ((**A**) 10 wt %, (**B**) 25 wt %, and (**C**) 50 wt %) before UV irradiation (dotted line), after UV irradiation (5 J/cm^2^) (dashed line) and after UV irradiation (5 J/cm^2^) and heating at 130 °C for 15 min (solid line).

The photo-deprotection of poly**1**–poly**4** was next carried out with triarylsulfonium salt CPI-110A as PAG under UV irradiation (5 J/cm^2^), followed by heating at 130 °C for 15 min. Triarylsulfonium salt decomposes upon UV irradiation to generate a Brønstead acid (HSbF_6_) (Scheme 6) [18]. Formation of *N*-H polyamide after removal of the *N*-alkoxybenzyl group was estimated by ATR spectroscopy. The effect of the PAG dose was first studied with poly**1** film. When 10 wt % of PAG was used, no difference in the ATR spectra between before and after UV irradiation and heating was observed (Figure 2A). On the other hand, when 25 and 50 wt % of PAG was introduced into poly**1** film, absorption due to amide NH appeared at 3400 cm^−1^ after UV irradiation and heating (Figure 2B,C). Accordingly, it turned out that more than 25 wt % of PAG was necessary for the removal of the methoxybenzyl group from poly**1**.

**Figure 3:**
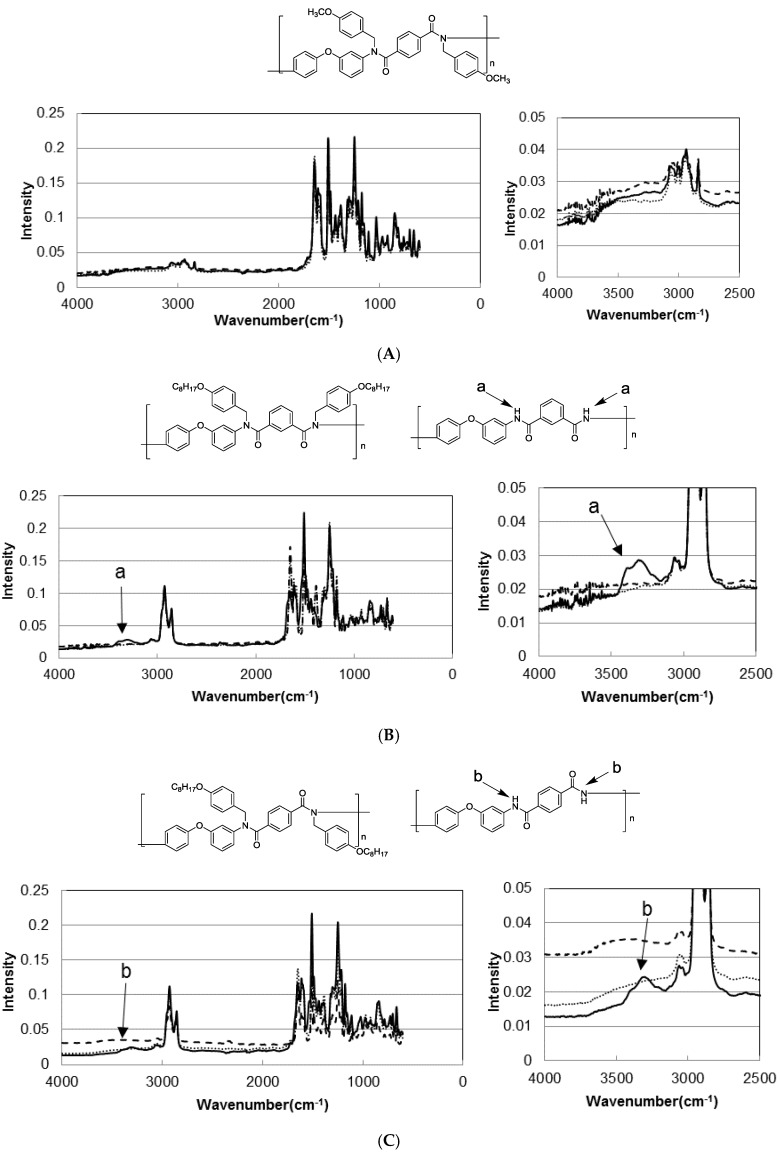
ATR spectrum of photosensitive film of poly**2**–poly**4** with 10 wt % of PAG before UV irradiation (dashed line), after UV irradiation (5 J/cm^2^) (dashed line) and after UV irradiation (5 J/cm^2^) and heating at 130 °C for 15 min (solid line): (**A**) poly2, (**B**) poly3, and (**C**) poly4.

To examine whether this was the case for all polyamides, however, photodeprotection of poly**2**–poly**4** was carried out with 10 wt % of PAG under the same conditions. Poly**2,** having a methoxybenzyl group, gave similar results to poly**1** (Figure 3A), whereas poly**3** and poly**4,** having an octyloxybenzyl group, were converted to *N*-H polyamides (Figure 3B,C). In addition, deprotection of even poly**3** and poly**4** did not proceed when the films were not heated at 130 °C after irradiation; the Brønstead acid generated from PAG would not diffuse in the film if the film was not heated. It should be noted that photodeprotection of the *N*-alkoxybenzyl polyamide film with PAG was influenced by the nature of the alkoxy group in the *N*-protecting group, but not by the dicarboxylic acid moiety: IPC or TPC, although no difference was observed in the deprotection of the polymer solution with TFA. This different photodeprotection behavior of the polyamide film depending upon the difference of the alkoxybenzyl group is presumably accounted for by different polymer topology between polyamide having the methoxybenzyl group (poly**1** and poly**2**) and polyamide having the octyloxybenzyl group (poly**3** and poly**4**). Thus, the former two are linear polymers with an amino group at both ends, whereas the latter two are cyclic polymers, as shown in Figure 1. Accordingly, the amino end groups in poly**1** and poly**2** might neutralize the acid species generated from PAG, when the concentration of PAG in the polyamide film is low. Thus, control of polymer end structure is important for photodeprotection of *N-*alkoxybenzyl aromatic polyamide film containing low levels of PAG.

## 4. Conclusions

We have demonstrated that highly soluble *N*-alkoxybenzyl aromatic polyamides can serve as photosensitive materials through photodeprotection of the alkoxybenzyl group with PAG, affording *N*-H polyamides with low solubility. The requisite polyamides were prepared by means of polycondensation of *N-*alkoxybenzyl aromatic diamine and dicarboxylic acid dichloride in the presence of pyridine at room temperature. Poly**1** and poly**2**, which were prepared from *N*,*N*-bis(4-methoxybenzyl)-3,4′-DDE (**1**) with IPC and TPC, respectively, contained cyclic polymer and linear polymer with an amino group at both ends, whereas poly**3** and poly**4**, which were prepared from *N*,*N*-bis(4-octyloxybenzyl)-3,4′-DDE (**2**) with IPC and TPC, respectively, were all-cyclic polymers. Poly**1**–poly**4** showed high solubility in a variety of organic solvents, in contrast to the corresponding *N*-H polyamides, poly**5** and poly**6**. Photodeprotection of poly**1** and poly**2** with 10 wt % of PAG under 5 J/cm^2^ of UV irradiation, followed by heating at 130 °C for 15 min did not proceed, whereas poly**3** and poly**4** afforded the corresponding *N*-H polyamides under the same conditions. The terminal amino groups in poly**1** and poly**2** presumably suppressed diffusion of Brønstead acid (HSbF_6_) generated from PAG, although even poly**1** underwent photodeprotection with more than 25 wt % of PAG. Our results show that the nature of the polymer end groups is crucial for photodeprotection with a low dose of PAG. 

## Figures and Tables

**Table 1:** Polymerization of *N-*alkoxybenzyl aromatic diamine with dicarboxylic acid ^a^.

Entry	*N*-Alkoxybenzyl Aromatic Diamine	Dicarboxylic Acid Chloride	*N*-Alkoxybenzyl Aromatic Polyamide	Yield (%)	*M*_n_^b^	*M*_w_^b^	*M*_w_/*M*_n_^b^
1	**1**	IPC	poly**1**	94	8630	13,500	1.57
2	**1**	TPC	poly**2**	91	5200	7070	1.36
3	**2**	IPC	poly**3**	96	14,900	23,250	1.56
4	**2**	TPC	poly**4**	77	7250	9750	1.30

^a^ Polymerization of equimolar diamine and dicarboxylic acid chloride was carried out in the presence of 2.2 equiv. of pyridine in NMP ([**1**]_0_ = [**2**]_0_ = 1.1 M) at room temperature for 2 days; ^b^ Determined by GPC based on polystyrene standards (eluent: THF).

**Table 2:**
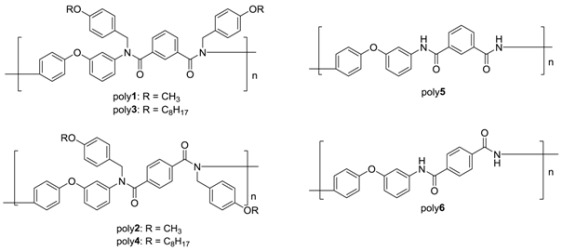
Solubility of poly**1**–**6** ^a^.

Solvent	poly1	poly2	poly3	poly4	poly5	poly6
Hexane	−	−	−	−	−	−
Acetone	+	+	+	+	−	−
Ethyl acetate	+	+	+	+	−	−
CH_2_Cl_2_	+	+	+	+	−	−
Chloroform	+	+	+	+	−	−
Methanol	−	−	−	−	−	−
DMF	+	+	+	+	+	+
NMP	+	+	+	+	+	+
DMSO	+	+	+	+	+	+

^a^ +, soluble; −, insoluble.

## References

[1] Kanki T., Ikeda J., Kobayashi Y., Suda S., Nakata Y., Nakamura T. Development of Highly Reliable Cu Wiring of l/s = 1/1mm for Chip to Chip Interconnection. Proceedings of the 2012 IEEE International Interconnect Technology Conference.

[2] Hu D.-C., Lin P., Chen Y.H. (2015). Fine trace substrate with 2 mm fine line for advanced package. Trans. Jpn. Inst. Electron. Packag..

[3] Huemoeller R., Rusli S., Chiang S., Chen T.Y., Baron D., Brandt L., Roelfs B. Unveiling the Next generation in substrate technology. Proceedings of the Pan Pacific Conference.

[4] Ghosh M. (1996). Polyimides: Fundamentals and Applications.

[5] Rubner R. (1990). Photoreactive polymers for electronics. Adv. Mater..

[6] Iwashita K., Katoh T., Nakamura A., Murakami Y., Iwasaki T., Sugimasa Y., Nunoshige J., Nakano H. (2015). Study of adhesion properties of Cu on photosensitive insulation film for next generation packaging. J. Photopolym. Sci. Technol..

[7] Mark H.F., Atlas S.M., Ogata N. (1962). Aromatic polyamide. J. Polym. Sci..

[8] Dine-Hart R.A., Moore B.J.C., Wright W.W. (1964). Aromatic polyamides. J. Polym. Sci. B.

[9] Harschnitz R., Heather P., Derks W., van Leeuwendal R. (1990). Polyamide 46—A design material for demanding engineering applications. Kunstst. Ger. Plast..

[10] Gijsman P., Tummers D., Janssen K. (1995). Differences and similarities in the thermooxidative degradation of polyamide 46 and 66. Polym. Degrad. Stab..

[11] Janssen K., Gijsman P., Tummers D. (1995). Mechanistic aspects of the stabilization of polyamides by combinations of metal and halogen salts. Polym. Degrad. Stab..

[12] Arai T., Ueno T., Kajiya T., Ishikawa T., Takeda K. (2007). Study on thermal stability and chemical structure of polyamide blended with small amount of Cu. Kobunshi Ronbunshu.

[13] Ohtsuka K., Matsumoto A., Kimura H., Saito M., Yamano K. (2008). Improvement of adhesive property of diallyl phthalate resin modified with dimeric acid polyamide. J. Netw. Polym. Jpn..

[14] Yokozawa T., Ogawa M., Sekino A., Sugi R., Yokoyama A. (2002). Chain-growth polycondensation for well-defined aramide. Synthesis of unprecedented block copolymer containing aramide with low polydispersity. J. Am. Chem. Soc..

[15] Ohishi T., Sugi R., Yokoyama A., Yokozawa T. (2006). A variety of poly(*m*-benzamide)s with low polydispersities from inductive effect-assisted chain-growth polycondensation. J. Polym. Sci. A.

[16] Kricheldorf H.R., Schwarz G. (2003). Cyclic polymers by kinetically controlled step-growth polymerization. Macromol. Rapid Commun..

[17] Kricheldorf H.R. (2009). Simultaneous chain-growth and step-growth polymerization—A new route to cyclic polymers. Macromol. Rapid Commun..

[18] Tsuda Y. (2016). Surface wettability controllable polyimides by uv light irradiation for printed electronics. J. Photopolym. Sci. Technol..

